# Atrial Natriuretic Peptide Inhibited ABCA1/G1-dependent Cholesterol Efflux Related to Low HDL-C in Hypertensive Pregnant Patients

**DOI:** 10.3389/fphar.2021.715302

**Published:** 2021-07-28

**Authors:** Yubing Dong, Yi Lin, Wanyu Liu, Wei Zhang, Yinong Jiang, Wei Song

**Affiliations:** Department of Hypertension, The First Affiliated Hospital of Dalian Medical University, DaLian, China

**Keywords:** atrial natriuretic peptide, ATP-binding cassette transporter A1, ATP-binding cassette transporter G1, cholesterol efflux, hypertensive disorders of pregnancy

## Abstract

**Objective:** It has been reported that atrial natriuretic peptide (ANP) regulates lipid metabolism by stimulating adipocyte browning, lipolysis, and lipid oxidation, and by impacting the secretion of adipokines. In our previous study, we found that the plasma ANP concentration of hypertensive disorders of pregnancy (HDP) was significantly increased in comparison to that of normotensive pregnancy patients. Thus, this study’s objective was to investigate the lipid profile in patients with HDP and determine the effects of ANP on the cholesterol efflux in THP-1 macrophages.

**Methods:** A total of 265 HDP patients and 178 normotensive women as the control group were recruited. Clinical demographic characteristics and laboratory profile data were collected. Plasma total triglycerides (TGs), total cholesterol (TC), low-density cholesterol (LDL-C), and high-density cholesterol (HDL-C) were compared between the two groups. THP-1 monocytes were incubated with different concentrations of ANP. ATP-binding cassette transporter A1 (ABCA1) and ATP-binding cassette transporter G1 (ABCG1) mRNA and protein were evaluated. ABCA1- and ABCG1-mediated cholesterol efflux to apolipoprotein A-Ⅰ (apoA-Ⅰ) and HDL, respectively, were measured by green fluorescent labeled NBD cholesterol. Natriuretic peptide receptor A (NPR-A) siRNA and specific agonists of the peroxisome proliferator–activated receptor-γ (PPAR-γ) and liver X receptor α (LXRα) were studied to investigate the mechanism involved.

**Results:** Plasma TG, TC, LDL-C, and LDL-C/HDL-C were significantly increased, and HDL-C was significantly decreased in the HDP group in comparison to the control (all *p* < 0.001). ANP inhibited the expression of ABCA1 and ABCG1 at both the mRNA and protein levels in a dose-dependent manner. The functions of ABCA1- and ABCG1-mediated cholesterol efflux to apoA-I and HDL were significantly decreased. NPR-A siRNA further confirmed that ANP binding to its receptor inhibited ABCA1/G1 expression through the PPAR-γ/LXRα pathway.

**Conclusions:** ABCA1/G1 was inhibited by the stimulation of ANP when combined with NPR-A through the PPAR-γ/LXRα pathway in THP-1 macrophages. The ABCA1/G1-mediated cholesterol efflux was also impaired by the stimulation of ANP. This may provide a new explanation for the decreased level of HDL-C in HDP patients.

## Introduction

Hypertensive disorders of pregnancy (HDP) is one of the leading causes of placental abruption, stroke, multiple organ failure, disseminated intravascular coagulation, intrauterine growth retardation, and intrauterine death (Anonymous, 2020). Although the etiology is multifactorial, changes in metabolism are also related to the prognosis of HDP. Turgay Emet et al. found that plasma total cholesterol (TC), triglyceride (TG), and low-density lipoprotein cholesterol (LDL-C) levels were significantly increased as pregnancy progressed (Emet et al., 2013). The plasma TG, TC, and LDL-C levels were higher in preeclampsia patients in a study with a southwestern India population (Bhat et al., 2019). Furthermore, maternal plasma TG increase and high-density lipoprotein cholesterol (HDL-C) decrease at late-stage gestation were associated with macrosomia risk and preterm delivery (Wang et al., 2018; Niyaty et al., 2020).

Atrial natriuretic peptide (ANP), which belongs to the natriuretic peptide family, is a polypeptide hormone that is mainly synthesized, stored, and secreted by the heart (Nakagawa et al., 2019). ANP was first recognized by de Bold et al. when a rapid and potent natriuretic response to intravenous injection of the atrial myocardial extract was observed in rats (de Bold et al., 1981). Pro-ANP was hydrolyzed by serine protease corin and released the same amount of N-terminal pro-atrial natriuretic peptide (NT-pro-ANP) and ANP when the blood pressure and volume increased (Nakagawa et al., 2019). There are three kinds of natriuretic peptide receptors in mammals. Natriuretic peptide receptor-A (NPR-A) is the principal receptor for ANP and brain natriuretic peptide. Natriuretic peptide receptor-B is the principal receptor for C-type natriuretic peptide. Natriuretic peptide receptor-C acts as a clearance receptor when binding with ANP. ANP contributes to maintaining blood pressure and body fluid homeostasis through diuresis, vasodilation, and inhibition of the renin–angiotensin system (Nakagawa et al., 2019). ANP knockout in adult male mice induced salt-sensitive hypertension (John et al., 1995). NPR-A knockout caused hypertension in mice (Oliver et al., 1997), indicating that ANP binding to NPR-A plays an important role in the regulation of blood pressure. Our previous study showed that the plasma NT–pro-ANP level was also significantly increased in HDP patients (Lin et al., 2021).

Recently, several lines of evidence have suggested that ANP is involved in lipid metabolism in different ways. Gabriella Garruti et al. found that ANP was expressed in and secreted from subcutaneous and visceral adipose tissue and pre-adipocytes (Garruti et al., 2007). ANP inhibited the proliferation of human visceral mature adipocytes and pre-adipocytes cultured *in vitro* (Sarzani et al., 2008). ANP was also expressed in and secreted from brown adipocytes (Bae and Kim, 2019), which induced thermogenic action (Kimura et al., 2017), and promoted adipose tissue browning (Bordicchia et al., 2012). Meanwhile, ANP accelerated lipolysis by phosphorylating hormone-sensitive lipase *in vitro* and *in vivo* (Sengenès et al., 2003; Birkenfeld et al., 2005) and induced lipid oxidation in humans (Birkenfeld et al., 2008).

ATP-binding cassette transporter A1 and G1 (ABCA1/G1) were transmembrane proteins that mediated cholesterol efflux to apolipoprotein A-I (apoA-I) and HDL-C. It was the first and rate-limiting step of reverse cholesterol transport (RCT). ABCA1 deficiency in patients (known as Tangier’s disease) demonstrated a high incidence of early-onset cardiovascular disease because of the extremely low HDL-C levels (Bodzioch et al., 1999). Harmen Wiersma et al. found that the plasma HDL-C level was significantly reduced in ABCG1 knockout mice treated with a high-cholesterol diet (Wiersma et al., 2009).

Our previous study demonstrated that ANP was increased in HDP (Lin et al., 2021); however, whether it affects the lipid profile remains unknown. Therefore, this study aimed to investigate whether ANP is involved in cholesterol metabolism.

## Materials and Methods

### Study Subjects

A total of 265 patients with HDP were enrolled in the HDP group from April 2014 to April 2017 at First Affiliated Hospital of Dalian Medical University (Dalian, China). A total of 178 normotensive women with gestational age >20 weeks were enrolled at the same time as the control group. The registration number for this clinical registration study was ChiCTR-ROC-17011468. Ethical approval for the study was obtained from the Human Ethics Committee of First Affiliated Hospital of Dalian Medical University. All the patients provided written informed consent before participating in the study.

The diagnostic criteria of HDP were in accordance with the guidelines published by the European Society of Cardiology in 2018 (Regitz-Zagrosek et al. (2018)), including preexisting hypertension, gestational hypertension, preeclampsia, preexisting hypertension plus superimposed gestational hypertension with proteinuria, and antenatally unclassifiable hypertension. The exclusion criteria were 1) gestational age <20 weeks; 2) malignant tumor or cancer, immune system disease, blood system disease, taking glucocorticoid drugs within two weeks, and taking immunosuppressants; 3) acute or chronic kidney disease, and taking diuretics; 4) secondary hypertension, severe liver dysfunction, and diabetes diagnosed before pregnancy; and 5) hyperthyroidism or hypothyroidism.

### Clinical Assessments

The clinical data, including age, height, prepregnancy body mass index (BMI), history of hypertension, past medical history, and blood pressure, were recorded in each patient’s prenatal health records. We selected the maximum blood pressure in the medical history for grouping because some of the patients were taking antihypertensive drugs. Fasting blood samples were collected during hospitalization before delivery, and serum creatinine, TC, TG, HDL-C, and LDL-C were measured using the Hitachi 7170 automatic biochemical analyzer in our Clinical laboratory Center (WEI RIKANG Bioengineering Co. Ltd., China). Urine was collected for urinalysis. Prepregnancy BMI and estimated glomerular filtration rate (eGFR) were calculated according to the formula BMI = height (kg)/weight^2^ (cm^2^), and eGFR = (186 × Scr)^−1.154^ × age^−0.203^ × 0.742.

### THP-1 Culture and Treatment

Human THP-1 macrophages (Procell, Wuhan, China) were cultured in RPMI-1640 supplemented with 0.05 nM β-mercaptoethanol, and 10% fetal bovine serum in 5% CO_2_ at 37°C. The cells were differentiated into macrophages by incubation with 100 ng/ml phorbol myristate acetate for 72 h. ANP was dissolved in basic RPMI-1640 to obtain the required concentrations (ranging from 10^−9^ to 10^−5^ mmol/L). The macrophages were cultured in medium containing different concentrations of ANP for 72 h. Total mRNA and protein were extracted for ABCA1 and ABCG1 detection. Liver X receptor α (LXRα) agonist 22-(R)-OH-cholesterol and proliferator-activated receptor-γ (PPARγ) agonist rosiglitazone were used to detect whether LXRα and PPARγ were involved in ABCA1 and ABCG1 expression mediated by ANP. NPR-A small interfering RNA (siRNA) was used to investigate whether NPR-A as a receptor was involved in ABCA1 and ABCG1 expression mediated by ANP. The macrophages were transfected with NPR-A siRNA and then coincubated with 10^−5^ mmol/L ANP for 72 h. Agonists of 22-(R)-OH-cholesterol (1 μM) and rosiglitazone (100 nM) were incubated with macrophages for another 48 h. Total protein was harvested for ABCA1, ABCG1, LXRα, and PPARγ detection.

### Quantitative Real-Time PCR (qRT-PCR)

Total RNA was extracted from the cells using TRIZOL reagent (BioTeke, Beijing, China) and then converted into cDNA by M-MLV reverse transcriptase (BioTeke, Beijing, China). The resulting cDNAs were subjected to qRT-PCR with SYBR™ green detection chemistry on an Exicycler™ real-time PCR system (Bioneer, Daejeon, Korea).

The following sequences of the real-time PCR primers were used: ABCA1 forward, 5′-TCA​CCA​CTT​CGG​TCT​CC-3′ and reverse 5′- CCA​CCT​TCA​TCC​CAT​CT-3′; ABCG1 forward, 5′-GGG​TCG​CTC​CAT​CAT​TT-3′ and reverse 5′- TGT​GGT​AGG​TTG​GGC​AGT-3′; β-actin forward, 5′-CAC​TGT​GCC​CAT​CTA​CGA​GG-3′, and reverse 5′- TAA​TGT​CAC​GCA​CGA​TTT​CC. The specificity of all the PCR products was assessed using the melting curve analysis. Relative gene expression was analyzed using the 2^−ΔΔCt^ method and normalized against β-actin as the internal control.

### Immunoblotting

The protein samples were loaded onto an 8% or 11% sodium dodecyl sulfate–polyacrylamide gel electrophoresis (SDS-PAGE) system for electrophoresis and blotting. The following antibodies were used: rabbit anti-ABCA1, anti-ABCG1 antibody (Sangon Biotech, Shanghai, China), rabbit anti–NPR-A, anti-LXRα antibody (ABclonal Technology, Wuhan, China), and rabbit anti-PPARγ, and β-actin antibody (Wanleibio, Shenyang, China). After incubation with the horseradish peroxidase–conjugated secondary antibody, the protein bands were visualized by enhanced chemiluminescence (ECL) (Wanleibio, Shenyang, China).

### Cellular Cholesterol Efflux Assays

THP-1 macrophages were incubated with 10^−5^ mol/L ANP for 72 h and then labeled with 5 μmol/L 22-NBD-cholesterol for 4 h. Afterward, the 22-NBD cholesterol labeled cells were rinsed with phosphate-buffered saline (PBS) and incubated in the presence of apoA-Ⅰ (15 μg/ml) or HDL (50 μg/ml) for 4 h. The fluorescence intensity (FI) of the medium and lysate was measured at 469 nm wavelengths for excitation and 537 nm for emission using a multimode microplate reader (BioTek, Winooski, VT, United States). Finally, the percent efflux was calculated using the following equation: FI (efflux medium)/[FI (efflux medium) + FI (cell lysate)] × 100%.

### SiRNA Transfection

Specific siRNAs against NPR-A (sense, 5′- GGC​CGA​GUU​AUC​UAC​AUC​UTT-3′; antisense, 5′-AGA​UGU​AGA​UAA​CUC​GGC​CTT-3′), and scramble negative control siRNA (sense, 5′-UUC​UCC​GAA​CGU​GUC​ACG​UTT-3′; antisense, 5′-ACG​UGA​CAC​GUU​CGG​AGA​ATT-3′) were synthesized by Jintuosi Company (Wuhan, China). The macrophages were transfected with siRNA duplexes (100 pM final concentration) using Lipofectamine™ RNAiMAX reagent (Invitrogen, Carlsbad, CA, United States). After 48 h, Western blot analysis was performed to determine the transfection efficiency.

### Statistical Analysis

Data were represented as the mean ± standard deviation. Clinical data were analyzed using SPSS software, version 17.0 (SPSS Inc., Chicago, IL, United States). The independent *t*-test was used if the distribution was normal; otherwise, the Mann–Whitney *U* test was used. Experimental data were presented using GraphPad Prism 8 (GraphPad Software, San Diego, CA, United States). One-way ANOVA and two-way ANOVA were used. *p* < 0.05 was considered to be statistically significant difference.

## Results

### Demographic Characteristics of the Two Groups

There were no significant differences in age, height, or prepregnancy BMI between the HDP group and the control group (each *p* > 0.05). The creatinine was significantly higher in the HDP group than in the control group (*p* < 0.01). The eGFR was significantly lower in the HDP group than in the control group (*p* < 0.01) (Table 1). 

**TABLE 1:** Demographic characteristics of the two groups.

	Control group (*n* = 178)	HDP group (*n* = 265)	*p* Value
Age (year)	31.06 ± 4.13	31.36 ± 4.62	0.542
Height (cm)	162.99 ± 5.17	163.03 ± 5.26	0.882
Prepregnancy BMI(kg/m^2^)	25.11 ± 3.08	25.51 ± 4.22	0.778
Creatinine (umol/L)	44.82 ± 0.59	51.98 ± 0.79	＜0.01^a^
eGFR (mL/min×1.73 m^2^)	140.77 ± 1.83	124.03 ± 1.73	＜0.01^a^

HDP, hypertensive disorders of pregnancy; BMI, body mass index; eGFR, estimated glomerular filtration rate.

a *p* < 0.01, compared with the control group.

### Maternal Plasma Lipid Profile of the Two Groups

**FIGURE 1:**
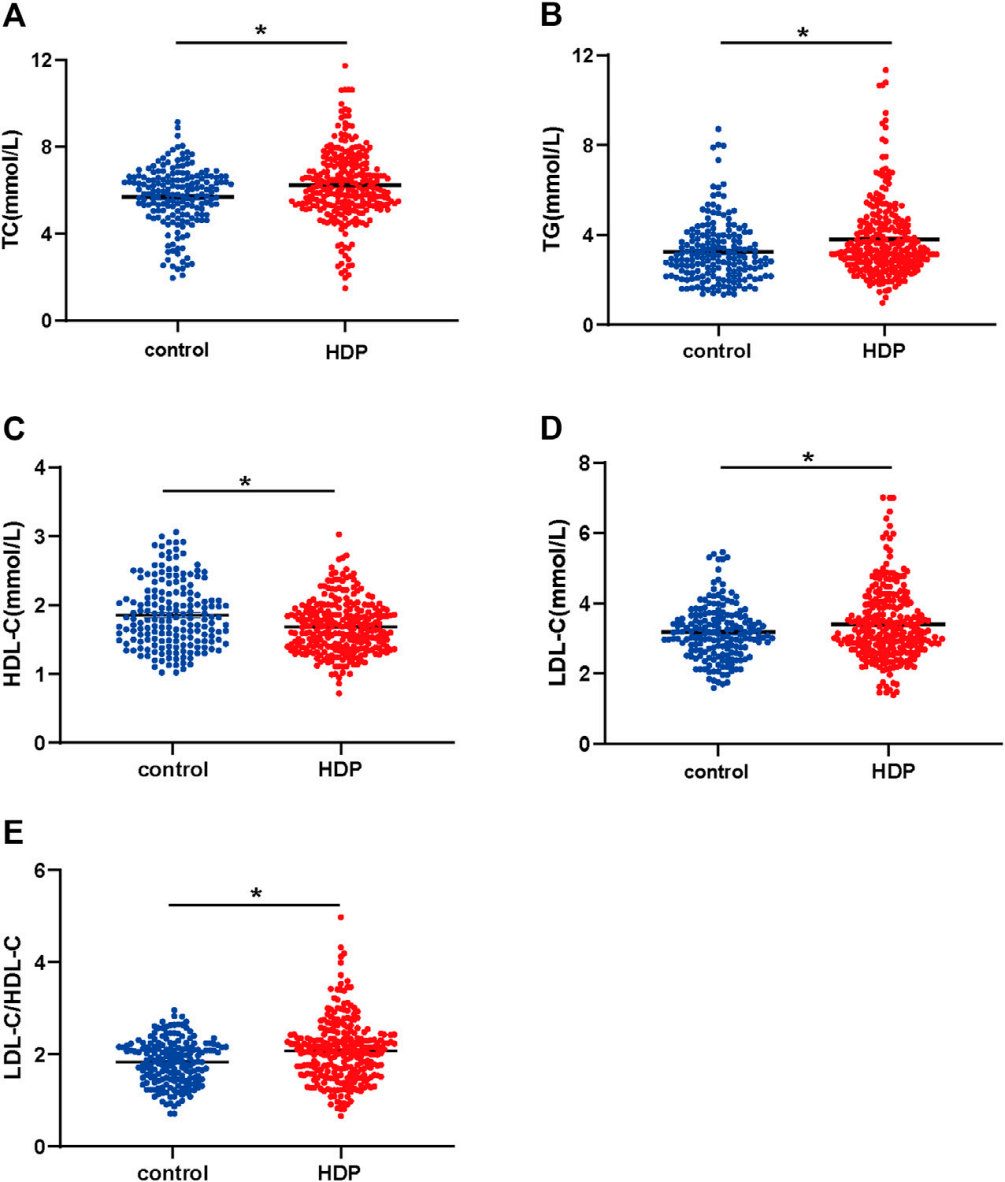
Maternal plasma lipid profile of the two groups. Plasma concentrations of TC (A), TG (B), LDL-C (C), HDL-C (D), and LDL-C/HDL-C ratio (E) were compared in the HDP group (*n* = 265) and the control group (*n* = 178). HDP, hypertensive disorders of pregnancy; TC, total cholesterol; TG, total triglycerides; HDL-C, high-density lipoprotein cholesterol; LDL-C, low-density lipoprotein cholesterol. *p < 0.01, compared with the control group.

The plasma concentration of TC was dramatically higher in the HDP group than that in the control group (6.23 ± 0.10 mmol/L vs. 5.69 ± 0.10 mmol/L, *p* < 0.01) (Figure 1A). The plasma concentration of TG was significantly increased in the HDP group in comparison to the control group (3.79 ± 0.11 mmol/L vs. 3.25 ± 0.10 mmol/L, *p* < 0.01) (Figure 1B). The plasma concentration of HDL-C was much lower in the HDP group than that in the control group (1.69 ± 0.02 mmol/L vs. 1.86 ± 0.04 mmol/L, *p* < 0.01) (Figure 1C). The plasma concentration of LDL-C was significantly higher in the HDP group than that in the control group (3.39 ± 1.03 mmol/L vs. 3.18 ± 0.78 mmol/L, *p* < 0.01) (Figure 1D). The plasma LDL-C/HDL-C ratio was markedly increased in the HDP group in comparison to the control group (2.07 ± 0.04 vs. 1.83 ± 0.04, *p* < 0.01) (Figure 1E).

### ABCA1 and ABCG1 Were Inhibited by the Stimulation of ANP in THP-1 Macrophages

**FIGURE 2:**
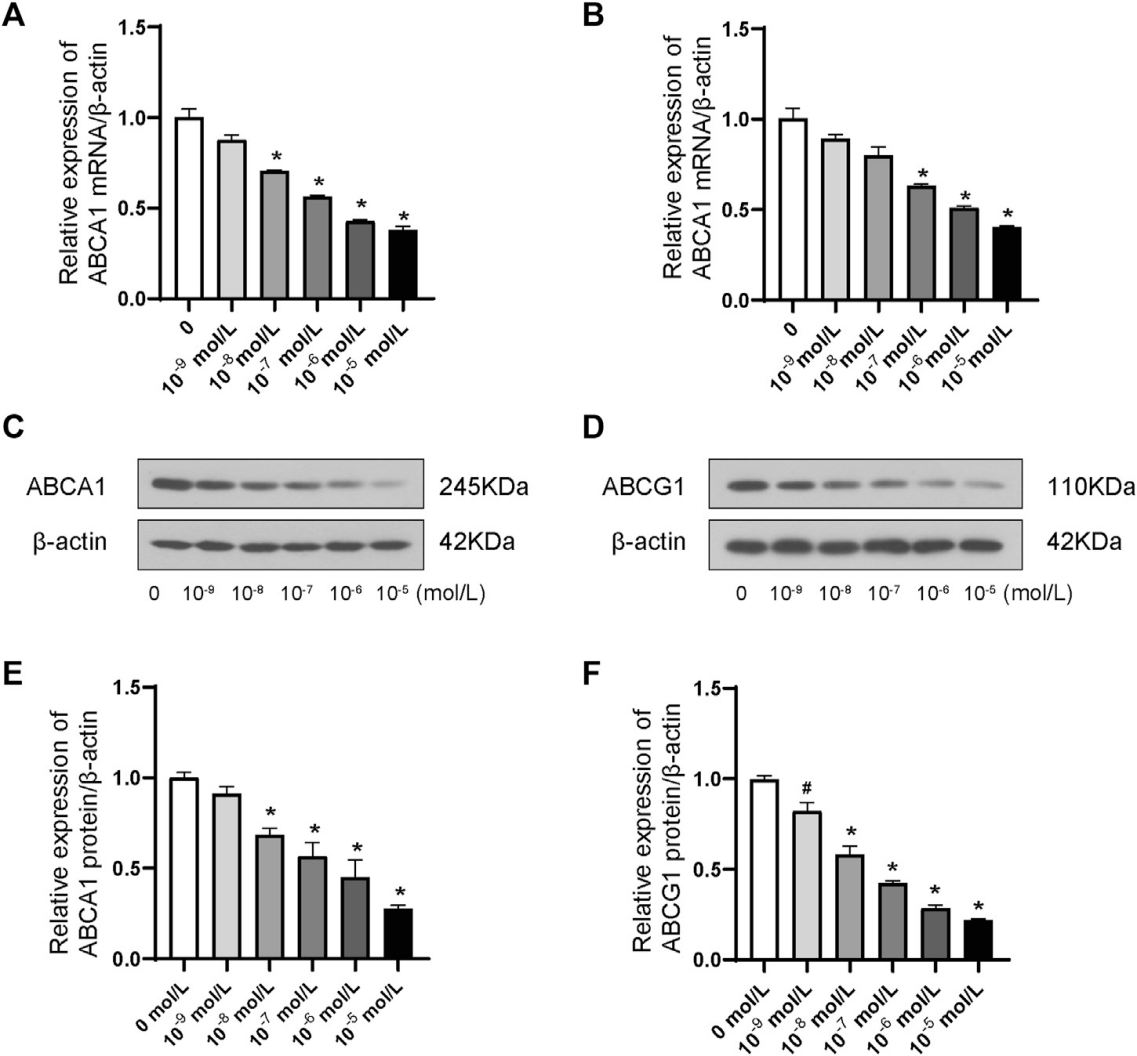
Dose-dependent effects of ANP downregulated ABCA1 and ABCG1 in THP-1 macrophages. A: ANP in concentrations ranging from 10^−9^ to 10^−5^ mol/L inhibited the ABCA1 mRNA expression in a dose-dependent manner (*n* = 3). B: ANP in concentrations ranging from 10^−9^ to 10^−5^ mol/L suppressed ABCG1 mRNA expression in a dose-dependent manner. C and E: ANP in concentrations ranging from 10^−9^ to 10^−5^ mol/L inhibited the ABCA1 protein expression in a dose-dependent manner. D and F: ANP in concentrations ranging from 10^−9^ to 10^−5^ mol/L suppressed ABCG1 protein expression in a dose-dependent manner. ^#^ *p* < 0.05, compared with the control group. *p < 0.01, compared with the control group.

ABCA1 and ABCG1 were dramatically inhibited in a dose-dependent manner at both the transcriptional (Figure 2A, B) and translational (Figure 2C–F) levels by the stimulation of ANP at concentrations ranging between 10^−9^ and 10^−5^ mol/L. Therefore, 10^−5^ mol/L was chosen as the optimal concentration in the subsequent experiments.

### ABCA1- and ABCG1-Mediated Cholesterol Efflux Was Impaired by ANP

**FIGURE 3:**
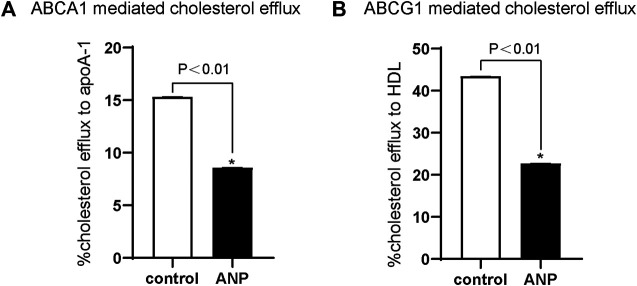
ANP inhibited ABCA1- and ABCG1-mediated cholesterol efflux. THP-1 cells were treated with the absence or presence of 10^−5^ mol/L ANP for 72 h. Cholesterol efflux was measured using apoA-Ⅰ (15 μg/ml) and HDL (50 μg/ml) as lipid acceptors. A: ABCA1-mediated cholesterol efflux to apoA-Ⅰ was attenuated in the ANP group. B: ABCG1-mediated cholesterol efflux to HDL was decreased in the ANP group. *p < 0.01, compared with the control group. Data are expressed as mean ± SEM of three independent experiments.

ABCA1-mediated cholesterol efflux to apoA-Ⅰ was markedly decreased by ANP treatment in comparison to the control group (*p* < 0.01) (Figure 3A). And ABCG1-mediated cholesterol efflux to HDL was also significantly inhibited by ANP treatment in comparison to the control group (*p* < 0.01) (Figure 3B).

### ANP Combined with NPR-A Inhibited ABCA1/G1 Through the PPARγ/Lxrα Pathway

**FIGURE 4:**
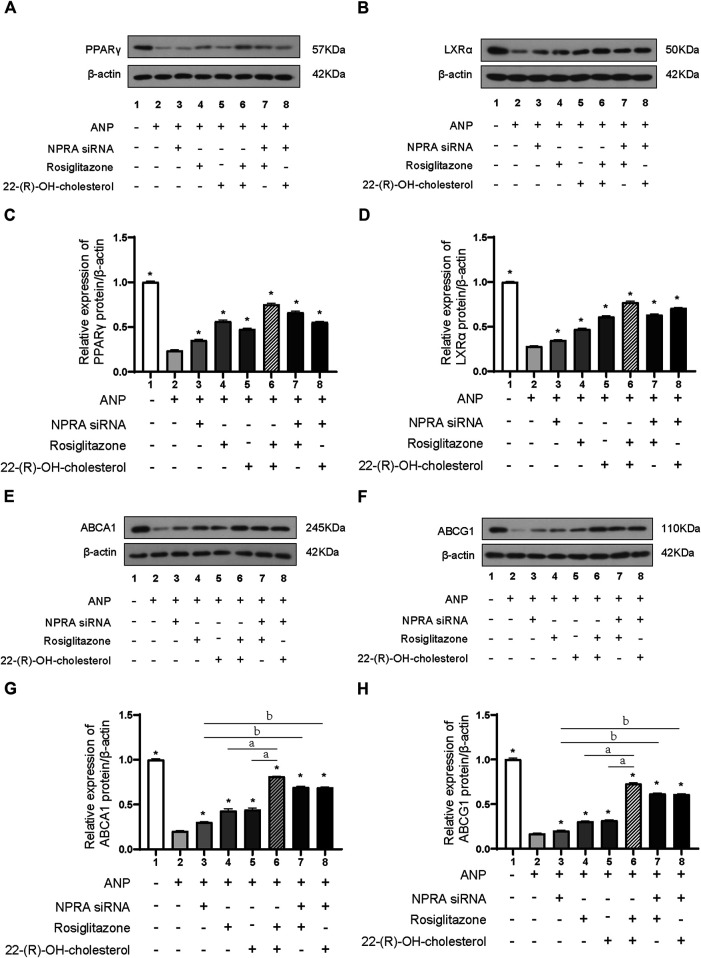
ANP inhibited the expression of ABCA1 and ABCG1 through the PPARγ/LXRα pathway. THP-1 cells were transfected with or without NPR-A siRNA and pretreated with or without ANP (10^−5^ mol/L) for 72h, and then incubated with or without rosiglitazone (100 nM) or 22-(R)-OH-cholesterol (1 μM) for another 24 h. The protein expressions of PPARγ **(A)**, LXRα **(B)**, ABCA1 (E), and ABCG1 (H) were examined by Western immunoblotting assays. β-actin was used as an endogenous control. The protein expressions of PPARγ **(C)**, LXRα **(D)**, ABCA1 **(G)**, and ABCG1 (H) were quantified by densitometric analysis of Western immunoblotting. *p < 0.01 vs. the ANP alone group. ^*a*^ *P* < 0.01 vs. the ANP + rosiglitazone+22-(R)-OH-cholesterol group. ^*b*^ *P* < 0.01 vs. the ANP + NPR-A siRNA group. Data are expressed as mean ± SEM of three independent experiments.

NPR-A expression was dramatically downregulated after NPR-A siRNA transfection (Supplementary Figure S1). PPARγ protein expression was decreased by ANP stimulation (*p* < 0.01) (Figures 4A, C lane 1 and lane 2). ANP also induced a significant decreased in LXRα protein expression (*p* < 0.01) (Figures 4B, D lane 1 and lane 2). PPARγ and LXRα protein expression was reversed by the treatment of NPR-A siRNA (lane 3), PPARγ agonist (lane 4), and LXRα agonist (lane 5) in comparison to ANP (lane 2) (*p* < 0.01) (Figures 4A–D). PPARγ and LXRα protein expression was further reversed by the combined treatment of NPR-A siRNA, PPARγ, and LXRα agonist (*p* < 0.01) (Figures 4A–D lanes 6–8). These results indicated ANP inhibited the PPARγ and LXRα expression by binding to NPR-A, and PPARγ and LXRα agonists can partially reverse the inhibition of PPARγ and LXRα induced by ANP.

ABCA1 and ABCG1 protein expression was partly reversed by the treatment of NPR-A siRNA (lane 3), PPARγ agonist (lane 4), and LXRα agonist (lane 5) in comparison to the ANP group (lane 2); they were almost totally reversed by the combination of PPARγ and LXRα agonists (lane 6) (*p* < 0.01) (Figures 4E–H). We inhibited NPR-A by siRNA and found that both the PPARγ agonist and the LXRα agonist abolished the inhibition of ABCA1 and ABCG1 induced by ANP (Figures 4E–H, lane 7 and lane 8). These results demonstrated that ABCA1 and ABCG1 were inhibited by ANP when combined with NPR-A through the PPARγ/LXRα pathway.

## Discussion

We found that the plasma concentrations of TG, TC, LDL-C, and LDL-C/HDL-C were increased, and the concentration of HDL-C was decreased in HDP patients. These results were consistent with a previous study of preeclampsia patients with a southwestern Indian population (Bhat et al., 2019). The plasma TG level in early pregnancy was positively associated with preeclampsia (Adank et al., 2019). These lipid profile alterations at late-stage gestation were associated with a neonatal adverse prognosis, such as macrosomia risk and preterm delivery (Wang et al., 2018; Niyaty et al., 2020). Furthermore, the maternal early-stage pregnancy plasma TC level was correlated to the TC and LDL-C levels of a mother’s 5- to 6-year-old offspring (van Lieshout et al., 2017). Therefore, alterations of the maternal blood lipid profile were related to the short- and long-term prognoses of the mother’s offspring. Thus, we should monitor the lipid profile in HDP patients. However, the mechanism of blood lipid profile changes in HDP is unclear.

Our previous study found that the plasma NT-proANP level in HDP patients was significantly increased (Lin et al., 2021). Dedoussis et al. found that ANP gene G664A polymorphism was associated with lower levels of apoA-Ⅰ and HDL-C in familial hypercholesterolemia patients (Dedoussis et al., 2006). HDL-C was found to be decreased in patients with coronary artery disease (CAD) when ANP was increased (Osajima et al., 2001). We found that NT-proANP levels were negatively correlated with HDL-C in the HDP group (*r* = −0.10, *p* < 0.05) (Supplementary Figure S2). Therefore, the increase in the plasma ANP found in the HDP patients may be related to low levels of HDL-C.

Hepatic ABCA1 plays an essential role in maintaining plasma HDL-C levels (Timmins et al., 2005). Mutations in the ABCA1 gene cause an extremely low plasma HDL-C level (Bodzioch et al., 1999). A study pointed out that the deficiency of ABCA1 in macrophages was probably reflected in its level in the liver, which resulted in low plasma HDL-C levels in CAD patients (Song et al., 2015). Therefore, we found that the deficiency of ABCA1 expression and function in macrophages induced by ANP stimulation may provide an explanation for the low plasma HDL-C level in HDP patients.

The effect of ABCG1 on the plasma HDL-C level remains controversial. Yan Xu et al. demonstrated that ABCG1 promoter region rs57137919 polymorphism caused the reduction of ABCG1 expression which promoted the occurrence of CAD but was not associated with the plasma HDL-C level (Xu et al., 2011). Harmen Wiersma et al. found that the plasma HDL-C level was significantly reduced in ABCG1 knockout mice treated with a high-cholesterol diet (Wiersma et al., 2009). Our study indicated that ANP inhibited the expression and function of ABCG1; however, more evidence is needed to determine if it is related to the low HDL-C level in HDP patients.

**FIGURE 5:**
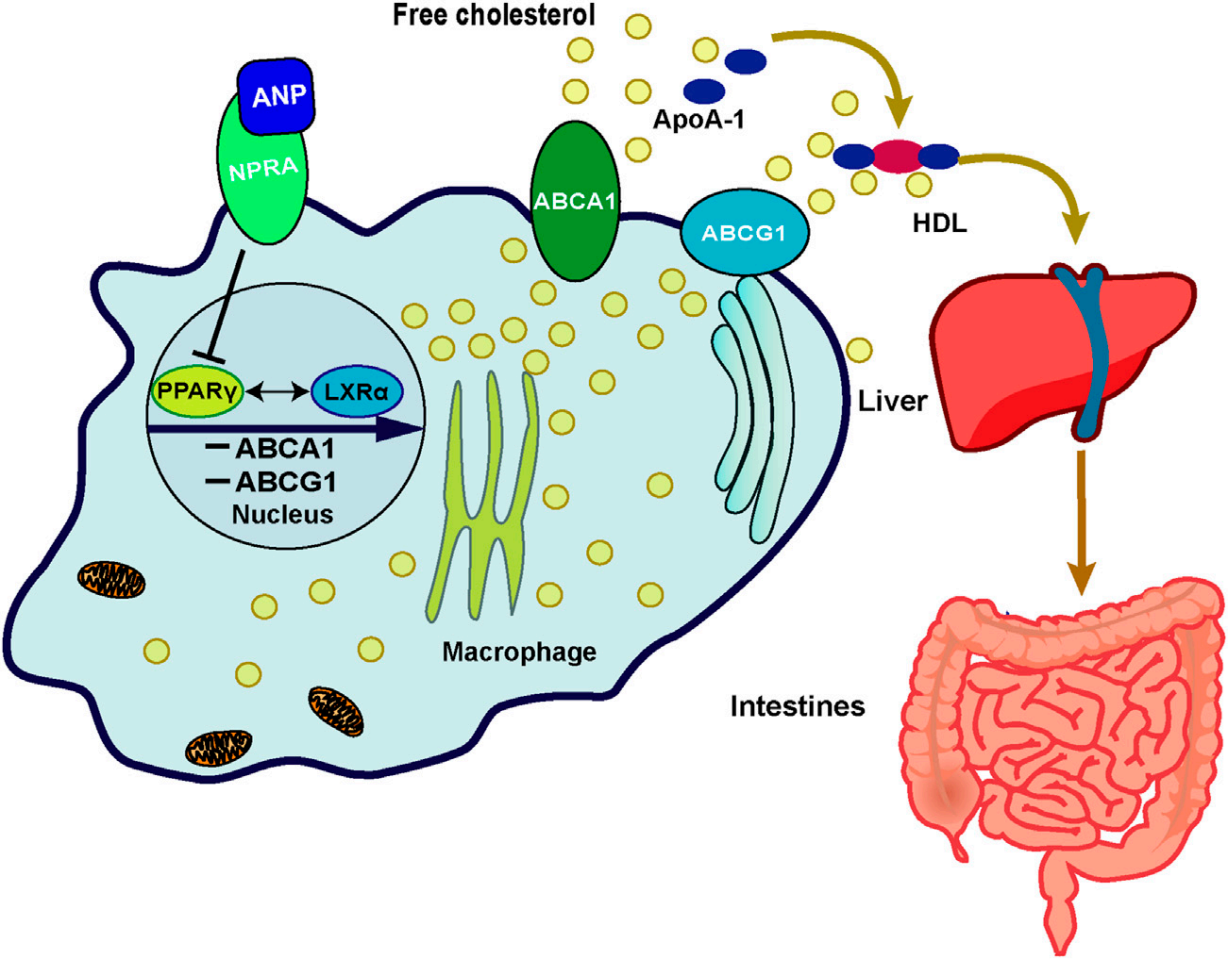
Schematic representation of the possible mechanism of ANP on the cholesterol efflux mediated by the PPARγ/LXRα–ABCA1/ABCG1 signaling pathway in macrophages. In the THP-1 derived macrophages, ANP inhibited the transcription of PPARγ and LXRα, then the expression of ABCA1 and ABCG1 was inhibited, thereby inhibiting cholesterol efflux from macrophages to apoA-I and HDL mediated by ABCA1 and ABCG1.

Although there may be other mechanisms, we found that the inhibition of ABCA1/G1 by ANP may be partially due to its binding to the NPR-A receptor. A previous study demonstrated that ANP had the highest affinity with NPR-A, which was consistent with our data (Nakagawa et al., 2019). Furthermore, we investigated the transcriptional regulation mechanism of ABCA1. Previous research showed that LXRα promoted RCT in human macrophages by upregulation of ABCA1 and ABCG1 expressions because four nucleotides (from −70 to −55 base pairs) located in the ABCA1 promoter were the binding sites to LXRα that promoted transcription (Costet et al., 2000). LXRα was also found to upregulate the transcription and expression of ABCG1 in macrophages (Ishibashi et al., 2013). Therefore, we thought that this key regulatory mechanism was also involved in the regulation of ANP on ABCA1. We found that the expression of PPARγ was enhanced by LXRα-specific agonists, and *vice versa*. This phenomenon was similar to the finding reported by Chawla et al., which showed that PPARγ stimulated the transcription of LXRα in macrophages (Chawla et al., 2001). Furthermore, there was other evidence which demonstrated that LXRα antagonist inhibited the transcription of PPARγ (Tsuboi et al., 2020). They proposed that LXRα and PPARγ formed a loop pathway to amplify the signals, which was further confirmed by our results. Thus, we concluded that ABCA1 and ABCG1 were inhibited by ANP when binding to its receptor NPR-A dependent on the PPARγ/LXRα pathway, which may be one of the mechanisms involved in the low HDL-C levels in HDP (Figure 5).

Andrew C. Li et al. found that the plasma LDL-C increased and the plasma HDL-C decreased in female mice but not in male mice after administration of PPARγ agonists (rosiglitazone or GW7845) to mice (Li et al., 2000). Therefore, the regulation of PPARγ on a lipid profile may be related to pregnancy in females. The safety of statins in pregnant women remains controversial. An obvious teratogenic effect was found in an animal study. There were case series studies which also demonstrated congenital anomalies associated with statin exposure, while some observational research works did not find an increased risk of congenital anomalies with statin therapy in pregnancy (Karalis et al., 2016). The possible reason for this was that the concentration of drugs used was higher for animals than for pregnant women. PPARγ agonists may become a new therapeutic target for clinical treatment for pregnant women.

Our study demonstrated for the first time that ANP binding to NPR-A inhibited ABCA1/ABCG1-mediated cholesterol efflux through the PPARγ/LXRα pathway. This mechanism may be involved in low HDL-C levels in HDP patients. These findings provide updated evidence that ANP may be involved in abnormal lipid metabolism in HDP.

## Data Availability

The original contributions presented in the study are included in the article/Supplementary Material; further inquiries can be directed to the corresponding author.
