# Supplementary material for: Atrial Natriuretic Peptide Inhibited ABCA1/G1-dependent Cholesterol Efflux Related to Low HDL-C in Hypertensive Pregnant Patients
Source: Front Pharmacol. 2021 Jul 28;12:715302. doi: 10.3389/fphar.2021.715302 (PMC8355588; doi:10.3389/fphar.2021.715302)
Supplement: Supplementary file 1 [file DataSheet1.PDF]

Supplemental Figure 1. NPRA siRNA transfection knockdown the expression of NPRA protein.

THP-1 derived macrophages were treated with three NPRA siRNA sequences and a scrambled siRNA. NPRA protein expression was downregulated after NPRA siRNA transfection as detected by Western blot, and NPRA siRNA-1 had the highest interference efficiency. NPRA siRNA-1 was chosen for subsequent experiments. *$P < 0.01$, compared with the control group. Data are expressed as mean ± SEM of three independent experiments.

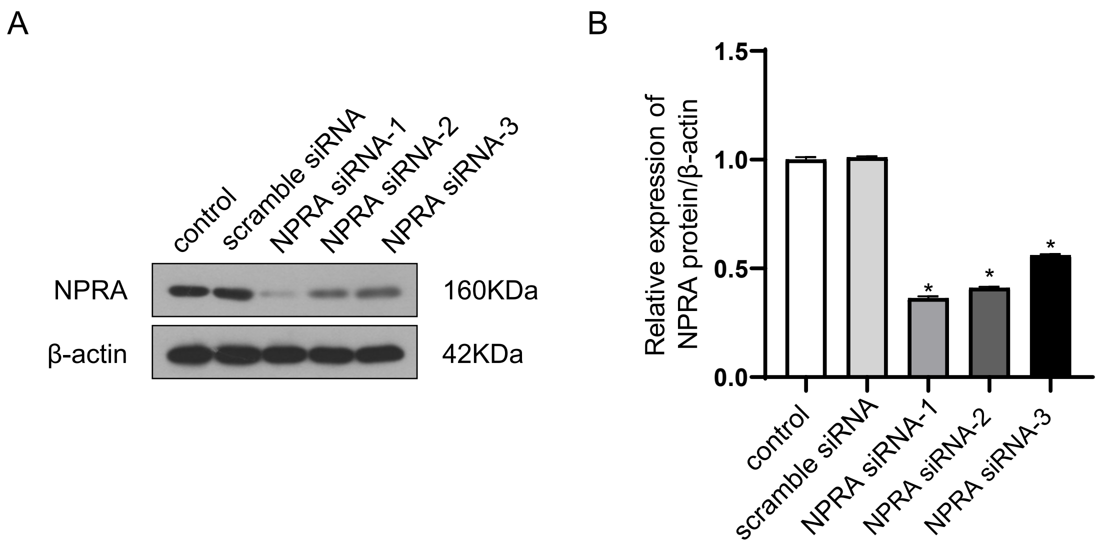
A
control
scramble siRNA
NPRA siRNA-1
NPRA siRNA-2
NPRA siRNA-3
NPRA
160KDa
β-actin
42KDa
B
Relative expression of NPRA protein/β-actin
1.5
1.0
0.5
0.0
*
*
*
control
scramble siRNA
NPRA siRNA-1
NPRA siRNA-2
NPRA siRNA-3

Supplemental Figure 2. Correlation of NT-proANP level with plasma HDL-C in the HDP group.

NT-proANP, N-terminal pro-atrial natriuretic peptide; HDL-C, high-density lipoprotein cholesterol.

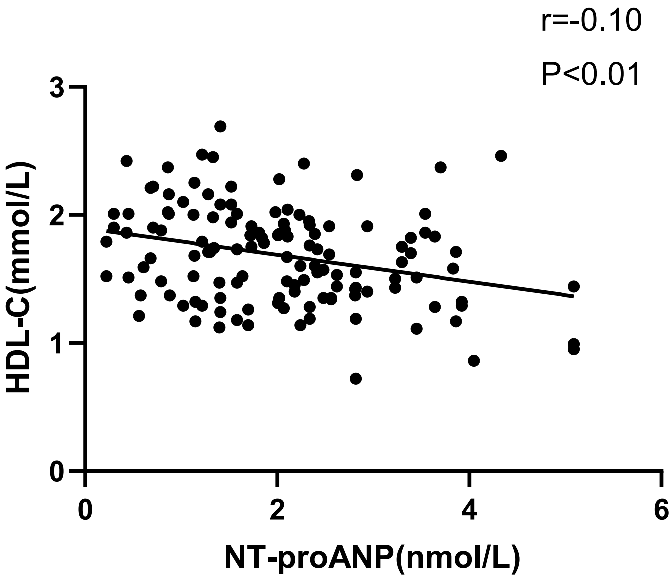

r=-0.10
P<0.01
3
2
1
0
0
2
4
6
HDL-C(mmol/L)
NT-proANP(nmol/L)
